# Editorial: Relevant technology adaptation for bio medical application and sustainability

**DOI:** 10.3389/fmedt.2023.1219073

**Published:** 2023-07-05

**Authors:** Anshuman Mishra, Manish Mishra

**Affiliations:** ^1^ Institute of Advanced Materials, IAAM, Ulrika, Sweden; ^2^ Department of Biomedical Sciences, Mercer University School of Medicine, Macon, GA, United States

**Keywords:** biomedical, personalized medicine, transcriptomics, healthcare management, sustainability

**Editorial on the Research Topic** Editorial: Relevant technology adaptation for bio medical application and sustainability

Recent advances in technologies have revolutionized the biomedical research and healthcare outcomes. The most promising areas of technological development in the biomedical field are within personalized medicine. In the personalized medicine approach, researchers identify an individual's genetic information via genomic sequencing and analysis to help physicians and patients to make better decisions in the prevention, diagnosis, and treatment of specific disease conditions (1). For example, personalized medicine can be used to tailor appropriate treatment or prevention for tumor/cancer taking into consideration of inter and intra-tumor variability in genes, immunological changes, and associated predisposing environmental factors, which will provide better outcomes and increased quality of life without disability (1). Further, advancements in wearable and portable devices can help to monitor vital signs in real-time and also enable healthcare providers to early detection and management of several medical conditions. These wearable and portable devices contain sensors which require special material design/synthesis strategies (if possible, biodegradable) to improve the sensitivity and specificity of desired detection parameters (2). Another area of innovation in biomedical technology is the development of implantable medical devices, like pacemakers, intraocular lenses, intrauterine contraceptives, artificial joints, and cochlear implants, which can be used in a variety of conditions, including heart disease, eye problems, birth control, partial or immovable joints, and hearing problems (3). These devices also need improved designs/materials so that they can reside inside the human body for a longer time without the requirement of replacement (2, 3). Furthermore, new imaging techniques like magnetic resonance imaging (MRI), positron emission tomography (PET), and computed tomography (CT) have greatly improved our ability to diagnose and monitor a variety of medical conditions, including cancer, heart disease, and neurological disorders, but their use is limited due to its size and cost (4). In the rise of telemedicine, rural clinics, and mobile health clinics, we need diagnostic technologies which can be portable or handheld and provide digital reports for the convenience of healthcare providers. These digital health technologies, like, mobile health apps, telemedicine platforms, and remote patient monitoring systems will significantly improve patient outcomes, reduce healthcare costs, and allow patients to receive care from the comfort of their own residential setup, while the same time it will enable healthcare providers to collect and analyze real-time data on patient health (1, 4). The rapid advances in the field of personalized medicine, wearable, handheld, or portable devices, implantable medical devices, and digital health technologies, offer unprecedented opportunities to improve human health and well-being. In the future, the aforesaid technologies will continue to evolve; and we can expect to see more economically sustainable innovative solutions for some of the world's most pressing health challenges (5, 6).

We are pleased to see the quality of the research submitted to our Research Topic *Relevant Technology Adaptation for BioMedical Application and Sustainability*, which finally contains two research and two review articles. These articles discussed the recent advancements and understandings in their respective fields. Herein, we provide a brief summary of these published articles and welcome the readers to read these publications and refer to their respective references for more details related to this Research Topic.

Nigam et al*.* emphasized the recent developments in fecal microbiota transplantation (FMT) treatment for gastrointestinal and extra-gastrointestinal disorders. MFT is a microbiota modification technique which reported to promote health in several diseases that cause microbial dysbiosis, like *recurrent* clostridium difficile infection (rCDI), irritable bowel syndrome/disease (IBS/IBD), and metabolic syndrome/insulin resistance. There are potential benefits of FMT treatment, which can be performed through colonoscopy, orogastric tube, enema, or supplementation of an oral capsule, but its effectiveness is variable among individuals and disease conditions. Several short-term clinical trials showed the beneficial impact of FMT, with no or limited adverse effects, but no long-term study is available for its effectiveness or adverse effects. Still, there are unresolved issues with the intricacy of implanted microbiota-host crosstalk due to several factors, like the genetics of microbes and individuals, the efficacy of metabolism, exposure to the gut environment (which is different in the various disease conditions), existing/modified microbial composition, and diet composition, which all may influence crosstalk. Despite the cost-effectiveness of FMT, other challenges still exist as fecal microbiota is a complex starting material. Therefore, there is a need for a well-defined selection criterion for a donor, requirement of large stool bank like blood bank to reduce the wastage of fecal materials, and standardization for patients who can have FMT. The future research needs to address a number of unresolved issues, including the timing of treatment, host aging, the right approach, the best strain for specific disease conditions, individualized prevention or therapy, and the use of “live” or “dead” microorganisms.

Wright et al*.* in their article compared the effectiveness of the cleanSURFACES® technology with the standard hospital protocols for disinfection on the high-touch surfaces in the two different hospital settings. In hospitals, the high-touch surfaces and contaminated equipment serve as reservoirs for the transmission of pathogens even following increased standard hygienic practices during the COVID-19 pandemic. They analyzed swabs samples from the high-touch surface areas using a meta-transcriptomic sequencing workflow (CSI-DxTM) to assess the microbial burden and re-contamination timeline. Their results confirmed that when cleanSURFACES® technology was coupled with routine hygienic practices, it effectively declined the microbial burden and microbial pathogens diversity on high-touch surfaces. So, if we target the high-touch areas with new technologies, like cleanSURFACES® technology along with standard hygiene practices in various clinical environments, it can reduce the prevalence of healthcare-associated infections (infections while receiving care at various healthcare facilities), which still remains one of the major public health challenges among patients, their visitors and health care workers.

Kankanige et al*.* explored the transcriptomics approach for accurately predicting druggable protein targets through bioinformatics algorithms tools. They explained the advantages and disadvantages of existing algorithms for gene expression, gene ontology, and protein interaction network prediction. They discussed the pros and cons of various gene enrichment algorithms analysis which is used for gene expressions data for identification of a particular gene/protein, which may share a common property with another gene/protein. Then they discussed the algorithms for protein interaction pathways which is the most important aspect of any cell's or tissue's functioning. These protein interaction changes during disease conditions and we can analyze if a drug can reverse or modify these protein interactions by using these tools. These tools are also useful in the click chemistry approach for the improvement of the drug by structural modifications. Presently, there is a gap in the existing algorithms that can combine all important analysis tools into a single unified and simplified algorithm package. If this gap can be filled with a new unified algorithm, it could accelerate the identification and prioritization of new drug targets and reduce the time and efforts associated with testing newly searched drug candidates for various diseases.

The fourth article by Etxeberria et al. is related to microfluidics technology development, which can be used in the form of Lab-on-a-chip (LoC) system for the development of analytical tools for pharmaceuticals, biomedical research, and clinical diagnostics. They explored the possibilities of downscaling the male and female 3-D printed Luer slip connector devices to reduce the carbon footprints by reducing the material used for chip-to-world interface in microfluidics (7). They designed, manufactured, and characterized seven variations of male and five variations of female connectors with varying dimensions to minimize the dead volumes while retaining their performance. Their specifically designed and proportionally decreased Luer Slip connectors (3 combinations) decline dead volumes without compromising the functionality of the connector, which may allow dense packaging or multiplexing in LoC devices.

At present, the adoption of relevant technologies for biomedical applications and their sustainability is crucial. The integration of advanced technologies holds the potential to drive profound changes and shape the future of healthcare in a sustainable and efficient manner. It is imperative that we recognize the significance of incorporating these innovations to address the pressing needs of the healthcare industry and ensure a sustainable and prosperous future.
